# Gut microbiota-mediated modulation of host amino acid availability and metabolism

**DOI:** 10.1080/19490976.2025.2552345

**Published:** 2025-08-28

**Authors:** Ziyi Han, Lingyang Zhao, Qilin Hu, Ifen Hung, Chunxue Liu, Shijie Liu, Xuehua Mei, Xiu Zeng, Peng Bin, Junhong Wang

**Affiliations:** a State Key Laboratory of Livestock and Poultry Breeding, College of Animal Science, South China Agricultural University, Guangzhou, China; b Anyou Biotechnology Group Co, LTD, Taicang, China; c Joint Laboratory of Functional Nutrition and Animal Health, Centree Bio-tech (Wuhan) Co., LTD, Wuhan, China; d National Center of Technology Innovation for Pigs, Chongqing, China

**Keywords:** Gut microbiota, amino acid metabolism, microbial homeostasis

## Abstract

The gut microbiota plays a crucial role in regulating host amino acid availability and metabolism through complex interactions with the host metabolic processes. This review summarizes three main mechanisms through which the microbiota influences host amino acid homeostasis. Firstly, gut microbiota actively competes for luminal amino acids derived from the diet and concurrently contributes to the host’s amino acid pool through *de novo* biosynthesis. Secondly, they modulate host intestinal hydrolases and amino acid transporters, thereby fine-tuning amino acid absorption and systemic bioavailability. Thirdly, microbially secreted factors, including diverse metabolites and extracellular vesicles, reprogram host amino acid metabolic pathways. The inherent inter-individual variations in microbial composition and metabolic capacity highlight the necessity of personalized therapeutic strategies targeting the microbiota. By summarizing current findings, this review aims to provide a deeper understanding of this dynamic interplay and to identify promising avenues for future investigations in the field.

## 1. Introduction

The gastrointestinal tract harbors a complex consortium of microorganisms collectively termed the gut microbiota, comprising over 1,000 bacterial species including archaea, eukaryotes, and viruses that encode 150-fold more unique genes than the human genome.^1–4^ This microbial ecosystem functions as a biochemical transformer, converting dietary substrates into metabolites that directly interface with both intestinal and systemic host physiology.^5,6^ Of particular relevance to host metabolism is the microbiota’s dual role in shaping amino acid (AA) availability: through the selective catabolism of luminal AAs and the *de novo* synthesis of microbial-derived AAs.^7,8^

Beyond their fundamental role as building blocks for proteins, AAs are key regulatory molecules involved in diverse physiological processes, including neurotransmitter synthesis and the regulation of glucose and lipid metabolism.^9,10^ Emerging evidence implicates gut microbiota-derived AA perturbations in various pathological conditions. For instance, in gastrointestinal infections, microbiota-driven depletion of luminal AAs by pathogens like *Clostridioides difficile* (*C. difficile*) and *Salmonella enterica* serovar Typhimurium (*S*. Typhimurium) can exacerbate dysbiosis.^11,12^ Conversely, certain gut commensals, like *Clostridium symbiosum* (*C. symbiosum*), alter AA availability to influence disease progression. A notable example is the excessive branched-chain amino acid (BCAA) production by *C. symbiosum*, which has been shown to promote cancer stemness via increasing cellular cholesterol synthesis in colonocytes.^13^

**Figure 1.**
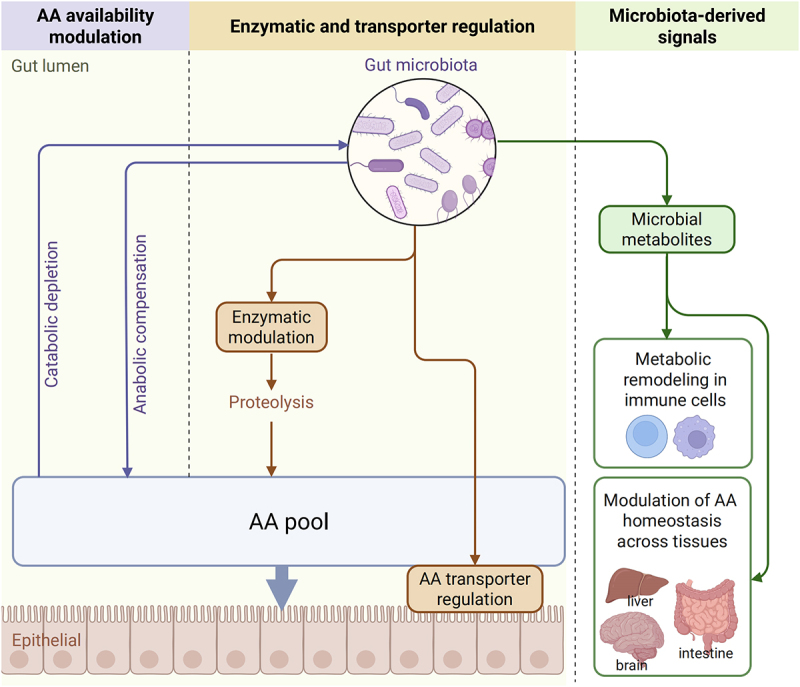
Gut microbiota-mediated modulation of host amino acid availability and metabolism.

Despite recent progress, the exact ways in which gut microbiota influence host AA metabolism remain incompletely understood. This review aims to synthesize current evidence regarding gut microbiota-mediated regulation of AA homeostasis, focusing on three interconnected mechanisms: (1) Microbial competition for dietary AAs versus their *de novo* AA synthesis; (2) Regulation of host intestinal hydrolases and AA transporters; and (3) Secretion of metabolites that reprogram host AA metabolic pathways (Figure 1). By summarizing current findings, this review aims to provide a comprehensive understanding of this area and to suggest directions for future studies.

This review summarizes three main mechanisms through which the microbiota influences host amino acid homeostasis: (1) Gut microbiota actively competes for luminal amino acids derived from the diet and concurrently contributes to the host’s amino acid pool through *de novo* biosynthesis (left). (2) They modulate host intestinal hydrolases and amino acid transporters, thereby fine-tuning amino acid absorption and systemic bioavailability (middle). (3) Microbially secreted factors, including diverse metabolites and extracellular vesicles, reprogram host amino acid metabolic pathways (right). (Created in https://BioRender.com.)

### 1.1. Gut microbiota directly modulates host AA availability

The gut microbiota dynamically shapes the intestinal AA pool through bidirectional metabolic processes, engaging in both catabolic depletion and anabolic synthesis of AAs (Table 1). These processes are highly context-dependent, influenced by factors such as microbial composition, host dietary patterns, and host AA requirements.

**Table 1.** Studies evaluating the role of microbiota as modulators in host AA availability.

Microbiota	Mechanism	References
Microbial catabolic depletion and anabolic compensation of AAs		
*C. difficile*	As a pathogen, it exacerbates dysbiosis by consuming luminal AAs; the avirulent strains competitively inhibit virulent strains by accelerating alanine/phenylalanine catabolism.	11, 22
*B. thetaiotaomicron*	A glutamate-fermenting commensal, its reduction leads to excessive glutamate consumption; supplementation with this strain reduces serum glutamate, increases lipolysis, and leads to weight loss.	27,28
*C. symbiosum*	It excessively produces BCAAs to promote cancer stemness by increasing cellular cholesterol synthesis in colonocytes.	13
*P. copri* and *B. vulgatus*	They elevate serum BCAAs in mice by synthesizing BCAAs and limit their own uptake, thereby enhancing host availability.	24
*F. prausnitzii*	It increases L-ornithine levels in Crohn’s disease patients, enhancing UST treatment sensitivity by interfering with IL-23 receptor signaling and inhibiting Th17 cell stabilization.	33
Microbial effect on luminal AA utilization via modulation of protease activity and AA transporters		
*Certain Lactobacillus and Bifidobacterium strains*	They produce microbial proteases that degrade immunogenic peptides (e.g., gluten and ATIs), increasing AA bioavailability and reducing inflammatory responses.	39
*P. clara* (strain ID: 1C4)	It utilizes a type IX secretion system to recruit host trypsin to the bacterial surface, inducing autolysis and thereby suppressing proteolytic activity.	41
*A. putredinis*	It produces β-glucuronidase (GUS) enzymes to catalyze the production of unconjugated bilirubin to inhibit host trypsin activity, maintaining protease homeostasis in the gut.	42
*L. reuteri*	It increases the expression of intestinal AA transporters (eg. *Slc6a19*, *Slc7a8*, *Slc15a1* and *Slc3a1*), elevating glutamine levels in the serum and brain, ameliorating neurobehavioral abnormalities in MS mice.	45
*B. uniformis*	It decreases the expression of AA transporters (*Slc6a19, Slc7a8, and Slc7a15*) and the serum glutamine levels to improve the mouse model of ASD-like behaviors and restore the E/I ratio in the brain.	46
*N. brasiliensis*	It downregulates the expression of LAT2, which mediates neutral AA transport.	47

#### 1.1.1. Microbial catabolic depletion

Luminal AA levels vary significantly between the small intestine and colon due to differences in digestive and absorptive processes. In the small intestine, dietary and endogenous proteins are digested by proteases and peptidases from the exocrine pancreas, resulting in peptides and free AAs at high levels. In contrast, the colon receives lower levels of AAs, primarily from undigested dietary proteins (estimated 4–12 g/day)^14^ and endogenous sources such as exfoliated epithelial cells and mucus, which are metabolized by the dense microbial population. Both pathobionts and commensals directly deplete luminal AAs via protein assimilation or catabolism to metabolites such as ammonia, short chain fatty acids (SCFAs) and indoles.^15–18^ Many gut microbes are auxotrophic for essential AAs, meaning they are unable to synthesize these nutrients and must obtain them from the environment. For instance, genomic analysis predicts that 63.9% of the human gut microbiota are auxotrophic for tryptophan (an essential AA for humans), while BCAA auxotrophy spans 40.1%–41.1%.^19^ This auxotrophy creates a competitive landscape where microbes and the host contend for the same limited resources. This competition is evident in experimental models; for instance, comparisons between specific pathogen free (SPF) mice and germ-free (GF) mice reveal lower levels of common protein-building AAs like proline, threonine, and asparagine in the cecum of SPF mouse compared to their GF counterparts, with concentration ratios of SPF/GF less than 0.1.^20,21^ This strongly suggests that microbial consumption significantly reduces AA availability for the host.

The availability of specific AAs can shape microbial competition, with auxotrophic bacteria relying on host or other microbial sources, while others synthesize amino acids *de novo*, affecting community dynamics. For example, the Human Reference Gut Microbiome (HRGM) catalog indicates that Actinobacteriota are more prone to lacking BCAA synthesis ability, often accompanied by the loss of genes for most enzymes required for the biosynthesis of corresponding AAs.^19^ This points to a more common demand from the nutritional environment provided by the host. Specific microbial strains can also outcompete others by rapidly depleting certain AAs. An illustrative example is an avirulent *C. difficile* isolate (ST1–75) that outcompetes its virulent strains (R20291) through accelerated alanine/phenylalanine catabolism in mice.^22^ This competitive advantage limits the growth of virulent R20291 through AA depletion, demonstrating how competition can alter microbial community dynamics and AA availability.

The AA preferences of gut microbiota are highly variable and are tailored to the specific ecological niche they inhabit within the gut. Although *Escherichia coli* (*E. coli*, a common inhabitant of the mammalian gut) preferentially consumes serine and threonine in the mono-colonized mouse models, the influence of *B. coccoides* on the availability of *E. coli*-preferred nutrients was substantial enough to alter *E. coli*’s evolutionary trajectory, leading it to utilize other carbon sources such as glucose and organic acids.^23^ The physiological environment also plays an important role in shaping the relationship between microbial communities and host AA metabolism. While *Bacteroides vulgatus* (*B.* *vulgatus*) was identified as a primary driver for increased levels of circulating BCAAs in mice,^24^ its enrichment in PMF-rich extract (PMFE)-treated high-fat diet (HFD) mice can also be associated with reduced BCAAs.^25^ This highlights the limitations of studying a single bacterial strain in isolation without considering the broader physiological context.

This competition can lead to unsuitable AA availability for the host, contributing to various health issues. For instance, in obese individuals, the gut microbiota exhibits enhanced AA biosynthetic functions (e.g., histidine and lysine) but impaired catabolism functions, resulting in AA imbalances.^26^ Notably, a marked reduction in *Bacteroides thetaiotaomicron* (*B. thetaiotaomicron*, a glutamate-fermenting commensal) can lead to excessive glutamate consumption, which is positively correlated with overweight in Chinese adults.^27^ Mice supplemented with this strain exhibited a 25% decrease in serum glutamate abundance, along with higher lipolysis and weight loss.^28^ Similarly, in type 1 diabetes (T1D) mice, the gut microbiota exhibits lower BCAA degradation ability, leading to excess BCAA intake from the gut to the liver and the heart. This in turn suppresses hepatic PPARα-FGF21 signaling and activates the cardiac mechanistic target of rapamycin (mTOR)/ L-type amino acid transporter 1 (LAT1) pathway, contributing to cardiac fibrosis and dysfunction.^29^

#### 1.1.2. Anabolic compensation mechanisms

In the large intestine, characterized by a higher density of microbial populations and extended residence times compared to the small intestine,^30^ gut microbiota can help replenish the luminal AA pool by degrading residual undigested proteins and synthesizing AAs.^31^ For instance, *Prevotella copri* (*P. copri*) and *B. vulgatus* can elevate serum BCAAs in mice by synthesizing BCAAs while limiting their own uptake, thereby enhancing host availability.^24^ Similarly, *Lactobacillus* and *Bifidobacterium* strains contribute to luminal pools of tryptophan and BCAAs via fermentation, demonstrating their role as AA contributors.^32^ Beyond proteinogenic AAs, microbial synthesis extends to non-protein AAs such as L-ornithine. *Faecalibacterium prausnitzii* (*F. prausnitzii*) elevates L-ornithine levels in Crohn’s disease (CD) patients undergoing ustekinumab (UST) therapy. This enhances UST treatment sensitivity and supports therapeutic efficacy in mouse models of CD by disrupting IL-23 receptor signaling and inhibiting Th17 cell stabilization.^33^ Such microbial synthesis can lead to a more efficient use of resources, recycling and synthesizing AAs that benefit both the microbial community and host, highlighting how microbiome encoded capabilities directly shape host AA homeostasis.

Luminal AA disturbance caused by gut microbiota leads to metabolic changes in the host liver. For example, gut microbiota-derived glutamine (elevated in antibiotic-treated mice) is metabolized to α-ketoglutarate (α-KG) in the liver, reprogramming macrophage metabolism via oxidative phosphorylation (OXPHOS), promoting M2 polarization and attenuating ischemia/reperfusion injury.^34^ Administration of *Akkermansia muciniphila* (*A. muciniphila*) in HFD and high-cholesterol (HFC) diet-induced obese mice elevates the liver levels of L-aspartate transported from the gut; this increase boosts mitochondrial oxidation and bile acid metabolism in the gut-liver axis and ameliorates oxidative stress-induced apoptosis in the gut.^35^

The balance between microbial AA consumption and production depends on the specific microbial community. Anabolic activities offset catabolic processes predominantly in certain ecological contexts, shaping host AA homeostasis in a community-specific manner.^7,36^ For example, when dietary AAs are scarce, competition may dominate as microbes vie for limited resources, potentially reducing AA availability for the host. Conversely, in nutrient-rich environments, cooperative interactions such as cross-feeding may become more prominent, allowing a more diverse and stable microbial community that enhances AA availability.^37^

### 1.2. Gut microbiota modulates AA absorption and transport through enzymatic and transporter regulation

The gut microbiota critically regulates AA absorption and transport via two interconnected mechanisms: (1) secretion of microbial proteases and modulation of host protease activity to affect luminal protein digestion and (2) regulation of AA transporter expression and activity (Table 1). These processes affect the bioavailability of AAs for host absorption and systemic metabolic outcomes.

#### 1.2.1. Gut microbiota affect luminal protein digestion via modulation of protease activity

**Figure 2.**
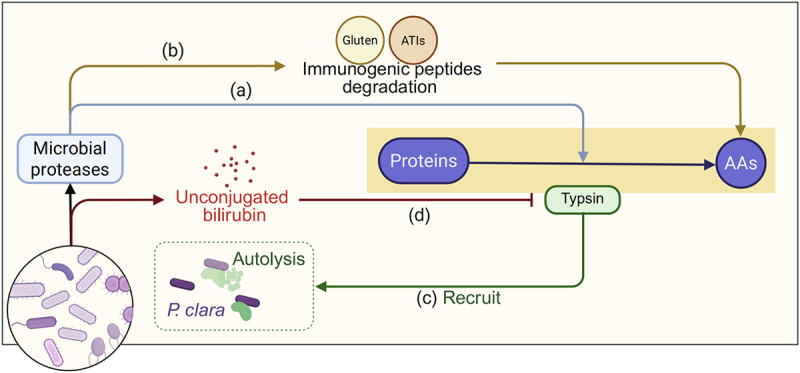
Gut microbiota affect luminal protein digestion via modulation of protease activity.

Gut microbiota can contribute to protein digestion by secreting proteases (e.g., extracellular peptidases and gluten-degrading enzymes) into the host lumen that hydrolyze dietary proteins into absorbable AAs and peptides (Figure 2(a)). These microbial proteases belong to classes analogous to human enzymes, with the notable addition of glutamic proteases – a class not found in mammals. The most abundant bacterial proteolytic enzymes are serine (39.2%), metalloproteases (35.9%), and cysteine (16.5%) proteases.^38^ Critically, microbial proteases hydrolyze undigested host proteins, thereby modulating their immunogenicity. For example, microbial proteases from certain *Lactobacillus* and *Bifidobacterium* strains can degrade dietary immunogenic peptides like gluten and amylase-trypsin inhibitors (ATIs) in mice, enhancing AA bioavailability while suppressing inflammatory responses (Figure 2(b)).^39^ This mechanism highlights its therapeutic potential for difficult-to-digest dietary components.

Gut microbiota affect luminal protein digestion via various mechanisms, including: (a) secretion of proteases into host lumen that hydrolyze dietary proteins into absorbable AAs; (b) degradation of dietary immunogenic peptides like gluten and amylase-trypsin inhibitors (ATIs); (c) recruitment of trypsin to the bacterial surface, inducing trypsin autolysis (specific to P. clara, strain ID: 1C4); and (d) secretion of β-glucuronidase (GUS) enzymes to catalyze the production of unconjugated bilirubin to inhibit host trypsin activity. (Created in https://BioRender.com.)

Additionally, microbes modulate host protease activity to fine-tune protein digestion.^40^ Elevated trypsin activities in fecal samples from inflammatory bowel disease (IBD) patients and IL10-deficient colitogenic mice suggest microbial dysbiosis disrupts trypsin regulation. The commensal *Paraprevotella clara* (*P. clara*, strain ID: 1C4) addresses this imbalance by utilizing a type IX secretion system to recruit host trypsin to the bacterial surface, inducing autolysis and thereby suppressing proteolytic activity in mice (Figure 2(c)).^41^ This scavenging effect may limit AA release in specific intestinal niches. Furthermore, since increased gut proteolytic activity is a hallmark of irritable bowel syndrome (IBS), microbial β-glucuronidase (GUS) enzymes contribute to protease modulation by catalyzing the production of unconjugated bilirubin to inhibit host trypsin activity (Figure 2(d)) in murine models.^42^ These microbial influences on protease activity vary with community composition, highlighting the context-dependent nature of AA availability for host absorption.

However, dysregulated microbial protease activity mediates inflammatory damage in intestinal diseases. Though representing higher levels of protein hydrolysis and AA release, excessive microbial proteases in the distal intestine also become pathogenic, especially in patients with inflammatory bowel disease (IBD) and IBS.^38^ For instance, proteases from *B. vulgatus* correlate with the severity of ulcerative colitis (UC, a major subtype of IBD) in humans. Mechanistically, these proteases disrupt epithelial barrier integrity via cleavage of tight junction proteins (ZO-1 and occludin). Supporting this causal role, broad-spectrum protease inhibitors ameliorate *B. vulgatus*-induced epithelial barrier dysfunction and prevent colitis in *B. vulgatus* mono-colonized, IL10-deficient mice.^43^

#### 1.2.2. Gut microbiota affect intestinal AA transport

Following proteolytic processing, AAs are absorbed via specialized transporters whose expression and activity are modulated by the gut microbiota.^44^ Although microbial catabolism reduces the luminal AA pool, specific microbial taxa can enhance transporter-mediated uptake of the remaining AAs. For example, beneficial microbes can regulate intestinal AA transporters to improve pathological conditions. Supplementation with *Lactobacillus reuteri* (*L. reuteri*) increases the expression of intestinal AA transporters (eg. *Slc6a19*, *Slc7a8*, *Slc15a1* and *Slc3a1*), elevating glutamine levels in the serum and brain, and in turn ameliorating neurobehavioral abnormalities in maternally separated (MS) mice.^45^ Similarly, supplementation with *Bacteroides uniformis* (*B. uniformis*) has been proved to improve the mouse model of autism spectrum disorder (ASD)-like behaviors and restore the excitation/inhibition (E/I) ratio in the brain by decreasing intestinal AA transport (*Slc6a19, Slc7a8, and Slc7a15*) and the serum glutamine levels.^46^ Conversely, pathogenic infection can inhibit AA transport. *Nippostrongylus brasiliensis* (*N. brasiliensis*) infection in rats downregulates the L-type AA transporter 2 (LAT2), which mediates neutral AA transport, thereby impairing AA uptake in a pathogen-induced suppression mechanism.^47^

The regulation of intestinal environment parameters such as pH by intestinal microbes also influences AA transporter activity.^48^ Microbial metabolites, particularly SCFAs, further influence transporter activity by acidifying the intestinal lumen (pH 4.5–6.0), thereby optimizing H^+^-dependent AA transport for AAs like proline and beta-alanine.^49–53^ Tryptophan transport, mediated by both Na^+^- and H^+^-dependent systems, is particularly sensitive to these pH shifts, with microbial SCFA production enhancing uptake efficiency.^44^

Microbial regulation of proteases and AA transporters reveals a sophisticated layer of control over nutrient absorption. However, the precise mechanism by which gut microbes regulate AA transporters in intestinal epithelial cells remains incompletely known. It is confirmed that dysbiosis-induced barrier disruption (e.g., increased permeability) indirectly impairs transporter functions, to a certain extent.^54,55^ For example, the levels of activating transcription factor 4 (ATF4) were significantly decreased in the inflamed intestinal mucosa, and the expression of SLC1A5 (a glutamine transporter) was directly regulated by ATF4 in cell lines.^56^ Therefore, intestinal barrier function ensures efficient AA absorption.^57^ These findings emphasize the importance of maintaining gut health, as a balanced microbiota can optimize nutrient absorption, promoting overall metabolic well-being.

### 1.3. Microbial-derived signals regulate host intracellular AA metabolism

The gut microbiota and host engage in dynamic crosstalk through bioactive molecules including microbial components, metabolites, and extracellular vesicles (EVs), which collectively reprogram host AA metabolism, particularly in immune cells (e.g., macrophages).^58,59^ Host-derived signals (e.g., neuroendocrine factors and dietary EVs) actively shape microbial community composition and metabolic output, forming a regulatory circuit essential for AA homeostasis.

#### 1.3.1. Microbial components mediate AA metabolic remodeling in immune cells

**Figure 3.**
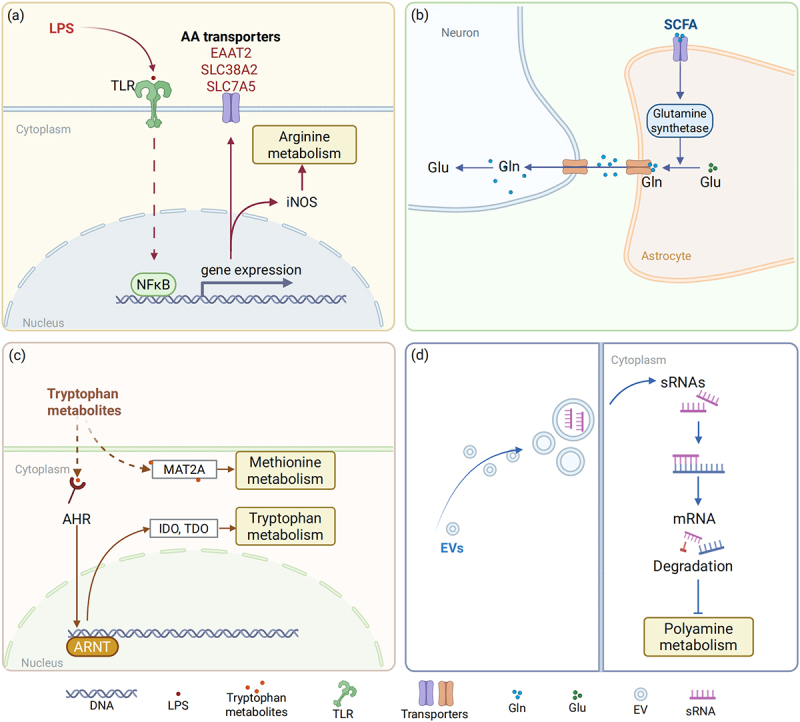
Microbial-derived signals affect host AA metabolism.

Microbial components, such as lipopolysaccharide (LPS) from Gram-negative bacteria, are pivotal in modulating AA metabolism in immune cells, particularly macrophages (Figure 3(a)). LPS-activated macrophages exhibit significant metabolic reprogramming, including altered AA utilization that produces greater amounts of glycine, glutamic acid, alanine, and histidine, while consuming serine and glutamine at higher rates.^60^ This metabolic shift is crucial for immune cell function and can influence the availability of specific AAs for other host processes. Mechanically, LPS interacts with host Toll-like receptors (TLRs), especially TLR4, triggering intracellular signaling cascades that lead to AA metabolic remodeling. This process involves the activation of nuclear factor-κB (NF-κB) through the recruitment of adaptor proteins like myeloid differentiation factor 88 (MyD88) following TLR4 dimerization.^61^ This pathway upregulates AA transporters, such as EAAT2, SLC38A2 and SLC7A5, in turn enhancing the uptake of AAs.^62,63^ Additionally, NF-κB modulates the mTOR signal transduction, a central regulator of cellular AA uptake and utilization, and induces enzymes like inducible nitric oxide synthase (iNOS), which metabolizes arginine.^64–66^ These metabolic shifts are crucial for immune cell function and can influence the availability of specific AAs for other host processes.

Microbial-derived signals, including LPS, SCFA, tryptophan metabolites and sRNAs, can affect host AA metabolism via distinct mechanisms: (a) In macrophages, bacterial LPS induces TLRs dimerization to activate NF-κB, which in turn upregulates the expression of AA transporters (EAAT2, SLC38A2 and SLC7A5), promoting the cellular uptake of AAs. The activation of NF-κB also promotes the generation of iNOS, thus enhancing host arginine metabolism. (b) Microbial SCFAs promote the glutamate-glutamine cycle in astrocyte-neuron cells through upregulating astrocyte glutamine synthetase to generate the Gln. Elevated Gln is shuttled to the neurons, where glutamine is reconverted to glutamate (Glu, glutamate; Gln, glutamine). (c) Microbial tryptophan metabolites activate AHR to enhance the expression of IDO and TDO involved in host tryptophan metabolism. They also interact with MAT2A to influence methionine metabolism. (d) Microbial sRNAs in EVs result in mRNA degradation of enzymes involved in polyamine synthesis of intestinal epithelial cells, thus downregulating host polyamine metabolism. (Created in https://BioRender.com.)

Similar mechanisms are observed in T cells, where the activation of TLR2/TLR1 or TLR2/TLR6 by microbial lipoproteins drives comparable AA metabolic changes. TLR2 activation in mouse CD4^+^ T cells upregulated SLC7A5 expression, enhancing leucine uptake to support T cell differentiation.^67,68^ The potential for therapeutic targeting of NF-κB to modulate AA metabolism in those conditions is promising, yet controversial due to its dual pro- and anti-inflammatory roles.^69^

#### 1.3.2. Microbial metabolites as regulators of host AA metabolism

Microbial metabolites represent a diverse class of signaling molecules that profoundly influence host AA homeostasis. SCFAs, such as butyrate, propionate, and acetate, produced by microbial fermentation of dietary fibers, are well-known examples.^70–72^ Butyrate, for instance, serves as a primary energy source for colonocytes and can inhibit histone deacetylase (HDAC) activity, thereby influencing gene expression related to AA metabolism in host cells.^73–75^ Notably, dietary SCFA supplementation in APPswe/PS1dE9 (APP/PS1) mice (a model for Alzheimer’s disease) can promote the glutamate-glutamine shuttle, maintaining AA balance in the brain, which protects against oxidative damage in neurons, with implications for therapies like Alzheimer’s disease (Figure 3(b)).^76^ Tryptophan metabolites, such as indole, and its derivatives [e.g. indole-3-propionic acid (IPA) and indole-3-acetic acid (IAA)], activate the aryl hydrocarbon receptor (AHR), exerting metabolic regulatory functions (Figure 3(c)).^77,78^ AHR activation, induces the expression of enzymes like indoleamine 2,3-dioxygenase (IDO) and tryptophan 2,3-dioxygenase (TDO), involved in tryptophan catabolism, creating a feedback loop that affects AA availability.^79^ This signaling also indirectly regulates microbial AA metabolism. For instance, AHR signaling enhances intestinal barrier function and promotes regulatory immune responses, creating an environment that supports specific microbial metabolic activities.^80^ IPA has been shown to influence methionine metabolism by interacting with methionine adenosyltransferase 2A (MAT2A) in murine inflammatory macrophages,^81^ highlighting the systemic impact of microbial metabolites, even at low concentrations.

#### 1.3.3. Extracellular vesicles (EVs) and precise adjustment in intracellular AA metabolism

Bacterial EVs, nano-sized membranous structures, are emerging as significant players in microbiota-host communication. These nanometer-sized vesicles contain a diverse cargo of bacterial components, including proteins, metabolites, and small RNAs (sRNAs), which can be delivered to host cells. Upon uptake by host cells, bacterial EVs can trigger various cellular processes, including AA metabolism.^82,83^ For example, bacterial EVs can deliver enzymes that directly modify host AAs or transport bacterial signaling molecules that reprogram host metabolic pathways.^84,85^ They can also influence immune cell activation and cytokine production, which in turn can indirectly affect AA metabolism in immune and other host cells.^86,87^ sRNAs, typically 50–250 nucleotides long, regulate gene expression at the posttranscriptional level.^88^ For example, in colistin-treated mice, *Lactobacillus murinus*-derived sRNAs in EVs downregulate colonic polyamine metabolism by targeting the expression of enzymes involved in polyamine synthesis, directly linking to AA metabolism (Figure 3(d)).^89^ While it is clear that bacterial EVs can affect host AA metabolism outcomes, studies emphasize the role in overall metabolic pathways (especially in pathological process) rather than direct AA modification. EVs from different bacterial species may have distinct metabolic functions, which require further comparison and verification.

#### 1.3.4. Host-derived signals reshape microbial metabolism

The host does not merely passively accept the influence of the gut microbiome, but actively regulates its gut microbiome through a variety of mechanisms. For example, a high dietary fiber diet is proved to increase microbial-derived SCFAs, which influence host immune cell functions (e.g., promoting Treg differentiation and IL-10 production), indirectly affecting microbial AA metabolism by altering the gut microbial composition.^80,90^ MicroRNA (miRNA), a type of sRNA, typically 20–25 nucleotides long, which is derived from the host and its dietary component, exhibits reciprocal regulation on modulating the gut microbiota composition. Host-derived fecal miRNAs (e.g., miR-1226-5p) enter bacteria [*Fusobacterium nucleatum* (*F. nucleatum*) and *E. coli*] via EVs, bind bacterial nucleic acids, and regulate growth-critical genes (e.g., *yegH* in *E. coli*). This reshapes microbiota composition and ameliorates DSS-induced colitis by repairing intestinal barrier integrity, indicating a miRNA-mediated inter-species gene regulation that facilitates host control of the gut microbiota.^91^ Diet-derived EVs similarly reprogram microbiota metabolism. For example, ginger exosome-like nanoparticles (GELNs) are selectively taken up by lactobacilli (e.g., *Lactobacillus rhamnosus* GG, LGG), targeting and suppressing the LGG monooxygenase gene *ycnE*, inhibiting its tryptophan metabolism. Furthermore, GELN miRNA ath-miR167a downregulates the LGG pilus protein SpaC, reducing bacterial invasion into intestinal epithelial cells and reshaping microbiota spatial distribution. This plant RNA-microbiota metabolite-host immune axis provides a novel strategy for dietary intervention in microbiota-related diseases.^92^ This host- and diet-mediated remodeling of microbiota composition and function indirectly reshapes the landscape of microbial AA metabolism and metabolite production within the gut.

## 2. Conclusions and future perspectives

The dual capacity of the gut microbiota to affect AA levels highlights it as a therapeutic target for cancers and metabolic diseases. One of the ways of utilization is to develop engineered probiotics (EPs) that degrade or produce specific AAs to manage symptoms. For example, Phenylketonuria (PKU) caused by a deficiency in phenylalanine hydroxylase (PAH), leads to high phenylalanine levels. *E. coli* Nissle 1917 with phenylalanine ammonia-lyase (PAL) has shown potential to reduce phenylalanine by 44.4% in PKU mice.^93^ For hyperammonemia, which results from ammonia metabolism disorders, *E. coli* Nissle 1917 has been engineered to convert lumen- and blood-derived ammonia to AAs like L-arginine or L-alanine.^94^ These studies highlight the potential of EPs to manage AA-related diseases by targeting specific metabolic pathways. The ability of certain gut microbes to affect AA levels as well as survive and thrive in tumors has been used to develop targeted cancer therapies that focus on the tumor microenvironment. As the availability of L-arginine in tumors is a key determinant of an efficient anti-tumor T cell response, an EP *E. coli* Nissle 1917 strain [with deletion of the arginine repressor gene (*ArgR*)] has been developed to increase intratumoural L-arginine concentrations and improve the efficacy of immunotherapy. The combined treatment with this L-Arg bacteria and anti-PD-L1 antibodies has successfully reduced tumor growth in MC38 tumors bearing mice (eradication rate in 74%) compared to those received anti-PD-L1 antibodies alone or combined with non-engineered strain (eradication rate in 44% and 39%, respectively).^95^ Some traditional Chinese medicines have also been proven to affect AA metabolism by influencing the gut microbiota, thereby improving pathological conditions. For instance, Xiaohua Funing Tang (XHFND) improves arginine metabolism imbalances by regulating the abundance of certain strains like Odoribacter and Bacteroides, suppressing Th17 cell-driven in gastritis models.^96^

Despite these advances, critical knowledge gaps persist: Firstly, although gut microbiota includes many species, research on non-bacterial organisms is less developed compared to bacteria, further studies are necessary to elucidate the roles of non-bacterial gut microbiota in host AA metabolism.^97^ Secondly, bioinformatic tools for annotating the potential functions of bacterial communities using genomic DNA or RNA sequences cannot fully reflect the active landscape of bacterial metabolism and functions, as most biological functions exert their effects at the protein level. Therefore, innovative technologies and methods (such as metagenome-informed metaproteomics^98,99^ need to be applied in the study of microbial host metabolism and diseases. Thirdly, current research on the interaction between host-microbiome-diet is largely relying on correlation analysis; however, microbial composition and metabolic output across individuals greatly limit the inference of causality, highlighting the need for biomarkers to predict AA metabolic outcomes.^100^

## 3. Compliance and ethics

Authors declare that they have no competing interests.

## 4. Declaration of generative AI and AI-assisted technologies in the writing process

During the preparation of this manuscript, the authors used ChatGPT-o1 and DeepSeek for the purposes of improving syntax and grammar checking. After using these tools, the authors reviewed and edited the content as needed and take full responsibility for the content of the publication.
